# Gyro-elastic beams for the vibration reduction of long flexural systems

**DOI:** 10.1098/rspa.2017.0136

**Published:** 2017-07-19

**Authors:** G. Carta, I. S. Jones, N. V. Movchan, A. B. Movchan, M. J. Nieves

**Affiliations:** 1 Liverpool John Moores University, Mechanical Engineering and Materials Research Centre, Liverpool, L3 3AF, UK; 2 University of Liverpool, Department of Mathematical Sciences, Liverpool, L69 7ZL, UK

**Keywords:** chiral system, gyro-elastic beams, Floquet–Bloch waves, vibration isolation

## Abstract

The paper presents a model of a chiral multi-structure incorporating gyro-elastic beams. Floquet–Bloch waves in periodic chiral systems are investigated in detail, with the emphasis on localization and the formation of standing waves. It is found that gyricity leads to low-frequency standing modes and generation of stop-bands. A design of an earthquake protection system is offered here, as an interesting application of vibration isolation. Theoretical results are accompanied by numerical simulations in the time-harmonic regime.

## 1. Introduction

Gyroscopic systems are always fascinating in their dynamic response and they are used in many applications in physics, engineering and aeronautics. In this paper, we show how to employ so-called gyrobeams (see, for example, [1]) in order to create low-frequency resonators, which can influence the behaviour of Floquet–Bloch waves in multi-structures subjected to dynamic excitations, such as earthquakes.

Many engineering structures (for example, bridges, pipelines and industrial warehouses) are designed as long chains of repetitive units, connected to each other by means of different types of joints. The dynamic study of such structures can be simplified by employing Floquet–Bloch theory, which reduces the problem to the analysis of a single unit cell with quasi-periodicity conditions. In this paper, we focus attention on flexural systems consisting of beam elements.

Periodic flexural systems have been extensively investigated in the last few decades [2–12]. In a recent work [13], it was proposed to use multi-scale high-contrast resonators to mitigate the vibrations of mechanical systems induced by earthquakes. These vibrations are generally difficult to suppress because they occur at low frequencies and are characterized by a wide spectrum. The range of frequencies in question for structures subjected to seismic loads is generally 0–30 Hz see [13]. The theoretical and practical challenge is to create, within the required frequency interval, ‘energy sinks’ where waves are channelled and thus diverted away from the main protected structure. Owing to their versatility, multi-scale high-contrast resonators can also be employed to protect randomly perturbed structures [14] from seismic waves. The incorporation of resonators into an elastic structure can lead to many interesting effects, such as generation of low-frequency standing waves [15], negative refraction [16,17], cloaking and filtering of seismic waves [18,19].

In this work, we propose a novel design of low-frequency resonators, which are not required to possess a high contrast mass to stiffness ratio. These resonators are beams with distributed gyricity, namely each volume element of the beam possesses additional angular momentum, as described in [1]. If the gyricity constant is sufficiently large, the structural element exhibits relatively low eigenfrequencies. Accordingly, gyro-elastic beams can be used as low-frequency resonators to reduce the vibrations of a structure, in a similar manner to that described in [13].

Gyroscopic systems have been analysed in conjunction with the motion of spinning spacecraft. The eigenvalue problems of linear gyroscopic systems were studied in [20,21], where the response of a rigid body connected to elastic components and subjected to an external force was also determined. The stability effect of gyroscopic forces and flutter instability in gyroscopic systems were discussed in [22–25].

The introduction of gyroscopic spinners into a structural model alters the properties of the structure, mainly due to the conferred chirality effects. Chirality is the property of an object whereby it cannot be superimposed onto its mirror image [26]. The dynamics of an elastic lattice connected to a system of rigid spinners was studied for the first time in [27], where it was shown how the dispersion surfaces of the lattice are modified by changing the parameters of the spinners. The phenomena of wave polarization and dynamic anisotropy occurring in lattices with rigid spinners were discussed in detail in [28], together with the frequency response of the lattice due to external harmonic excitations. The same model was used in [29,30] to generate one-way edge modes in an elastic lattice, where the time-reversal symmetry is broken by the gyroscopic term. Recently [31], a non-uniform system of gyroscopic spinners was designed to produce a very localized uni-directional wave pattern in an elastic lattice. It was shown that this wave pattern can be diverted, as in a deflecting elastic prism, by modifying the arrangement of the spinners inside the medium. This phenomenon was named DASER (Dynamic Amplification by means of Spinners in an Elastic Reticulated system).

The idea of ‘distributed gyricity’ was introduced in [1], where partial differential equations governing the motion of a gyro-elastic structure were derived and vibration modes were determined. This theory was developed further in successive papers [32,33]. Static and dynamic instabilities exhibited by gyro-elastic beams were discussed in [34].

The present paper is organized as follows. In §2, we give the equations of motion of a single beam with distributed gyricity and we determine the eigenfrequencies and eigenmodes for different values of the gyricity constant. In §3, we consider a structural frame, composed of a classical Euler–Bernoulli beam and a gyro-elastic beam, and we show how the eigenfrequencies of the frame are affected by gyricity. In §4, we determine the dispersion curves for a periodic system, consisting of an infinite number of repetitive frames containing gyro-elastic beams; in particular, we show that large stop-bands are generated when gyricity is present. In §5, we exploit the gyricity effect to reduce considerably the vibrations of a long structure, subjected to an external harmonic excitation. Finally, in §6 we provide concluding remarks.

## 2. Dynamics of a gyro-elastic beam

**Figure 1.**
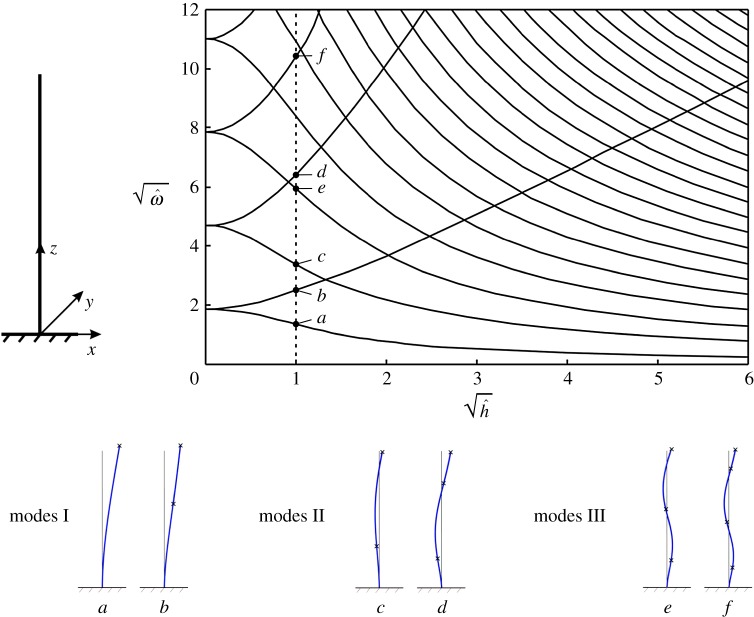
Normalized eigenfrequencies $\hat{\omega }$ of a clamped-free gyro-elastic beam for different values of the normalized gyricity constant $\hat{h}$. In the diagram, the square roots of $\hat{\omega }$ and $\hat{h}$ are plotted. Some of the eigenmodes are shown in the insets, where the small crosses indicate the positions of the inflection points. (Online version in colour.)

We consider an elastic Euler–Bernoulli beam of square cross section, which is clamped at one end and is free at the other end, as sketched in figure 1. The beam is characterized by a continuous distribution of gyricity, which is independent of the motion of the body and of the mass and stiffness distribution in the beam [1]; in addition, it is assumed for simplicity that gyricity is uniform along the beam length and is independent of time. Owing to this special feature, this structural element will be henceforth referred to as *gyro-elastic beam* or *gyrobeam*.

### (a) Equations of motion

In the transient regime, the governing equations describing the flexural motion of the gyrobeam without external loads and pre-stress are given by Yamanaka *et al*. [34]

2.1 $$\begin{array}{rl} & EJ\frac{\partial^{4}u(z,t)}{\partial z^{4}}+\rho A\frac{\partial^{2}u(z,t)}{\partial t^{2}}+h\frac{\partial^{3}v(z,t)}{\partial z^{2}\partial t}=0\\ \text{and}\; & EJ\frac{\partial^{4}v(z,t)}{\partial z^{4}}+\rho A\frac{\partial^{2}v(z,t)}{\partial t^{2}}-h\frac{\partial^{3}u(z,t)}{\partial z^{2}\partial t}=0, \end{array}\}$$

where *z* is the direction of the beam axis, *u* and *v* are the displacement components in the *x*- and *y*-directions, respectively, *t* is time, *E* is the Young’s modulus, *ρ* is the density, *A* and *J* are the cross-sectional area and second moment of area (identical in the two directions), respectively, and *h* is the gyricity constant. We note that the second moments of area in the *x*- and *y*-directions are identical, i.e. *J*_*x*_=*J*_*y*_=*J*, because we have considered a beam with a square cross section. For a rectangular cross section *J*_*x*_≠*J*_*y*_, and the equations (2.1) change accordingly.

The quantity *h* has the physical dimension of N s. The practical implementation of *h* depends on how a gyrobeam is modelled in engineering applications. In a different work, which is intended to provide a practical design for a gyrobeam, we have developed a concept based on the analysis of a multi-scale system of elastic links connecting gyroscopic spinners. It is assumed that the moments of inertia of each gyroscopic spinner with respect to the transverse axes are negligibly small in comparison with the moment of inertia *I* with respect to the spinner axis. The gyricity constant *h* is proved to be proportional to *I* and to the rate of spin *Ω* of individual gyroscopes, and it is inversely proportional to the distance *d* between neighbouring gyroscopes.

In the time-harmonic regime, the displacement components are expressed as *u*(*z*,*t*)=*U*(*z*) e^i*ωt*^ and *v*(*z*,*t*)=*V* (*z*) e^i*ωt*^, where *ω* is the radian frequency. In this case, equations (2.1) take the form

2.2 $$\begin{array}{rl} & EJ\frac{d^{4}U(z)}{dz^{4}}-\rho A\omega^{2}U(z)+ih\omega \frac{d^{2}V(z)}{dz^{2}}=0\\ \text{and}\; & EJ\frac{d^{4}V(z)}{dz^{4}}-\rho A\omega^{2}V(z)-ih\omega \frac{d^{2}U(z)}{dz^{2}}=0. \end{array}\}$$

The general solutions of (2.2) are given by

2.3 $$\begin{array}{rl} U(z) & =A_{1}\cos(\beta_{+}z)+A_{2}\sin(\beta_{+}z)+A_{3}\cosh(\beta_{-}z)+A_{4}\sinh(\beta_{-}z)\\ & \;+A_{5}\cos(\beta_{-}z)+A_{6}\sin(\beta_{-}z)+A_{7}\cosh(\beta_{+}z)+A_{8}\sinh(\beta_{+}z)\\ \text{and}\;V(z) & =i\{A_{1}\cos(\beta_{+}z)+A_{2}\sin(\beta_{+}z)+A_{3}\cosh(\beta_{-}z)+A_{4}\sinh(\beta_{-}z)\\ & \;-A_{5}\cos(\beta_{-}z)-A_{6}\sin(\beta_{-}z)-A_{7}\cosh(\beta_{+}z)-A_{8}\sinh(\beta_{+}z)\}, \end{array}\}$$

where *A*_*i*_ (1≤*i*≤8) are arbitrary constants and

2.4 $$\beta_{\pm }={\left(\frac{\omega [\mp h+\sqrt{h^{2}+4\rho EAJ}]}{2EJ}\right)}^{1/2}.$$

The expressions in (2.4) become equal to

$$\beta ={\left(\frac{\rho A\omega^{2}}{EJ}\right)}^{1/4}$$

when *h*=0 and consequently, in this case, the solutions *U*(*z*) and *V* (*z*) take the form

2.5 $$\begin{array}{rl} U(z) & =B_{1}\cos(\beta z)+B_{2}\sin(\beta z)+B_{3}\cosh(\beta z)+B_{4}\sinh(\beta z)\\ \text{and}\;V(z) & =C_{1}\cos(\beta z)+C_{2}\sin(\beta z)+C_{3}\cosh(\beta z)+C_{4}\sinh(\beta z), \end{array}\}$$

where *B*_*i*_, *C*_*i*_ (1≤*i*≤4) are arbitrary constants. The latter expressions are the displacement components of a classical Euler–Bernoulli beam under time-harmonic conditions.

The boundary conditions for a clamped-free gyrobeam are given by

2.6 $$\begin{array}{rl} U{|}_{z=0} & =0,\;V{|}_{z=0}=0,\;\phi_{x}{|}_{z=0}={\frac{dV}{dz}|}_{z=0}=0,\;\phi_{y}{|}_{z=0}={\frac{dU}{dz}|}_{z=0}=0,\\ M_{x}{|}_{z=L} & =EJ{\frac{d^{2}V}{dz^{2}}|}_{z=L}=0,\;M_{y}{|}_{z=L}=EJ{\frac{d^{2}U}{dz^{2}}|}_{z=L}=0,\\ T_{x}{|}_{z=L} & =EJ{\frac{d^{3}U}{dz^{3}}|}_{z=L}+ih\omega {\frac{dV}{dz}|}_{z=L}=0\\ \text{and}\;T_{y}{|}_{z=L} & =EJ{\frac{d^{3}V}{dz^{3}}|}_{z=L}-ih\omega {\frac{dU}{dz}|}_{z=L}=0, \end{array}\}$$

where *L* is the length of the gyrobeam, while *ϕ*_*j*_, *M*_*j*_ and *T*_*j*_ (*j*=*x*,*y*) are the rotations, bending moments and modified shear forces, respectively, with respect to the axes *x* and *y*. We note that the bending moments have the same expressions as for a classical Euler–Bernoulli beam, while the shear forces contain a term that depends on the gyricity constant *h* [34].

### (b) Eigenfrequency analysis

The eigenfrequencies and eigenmodes of the clamped-free gyrobeam are obtained by solving the spectral problem (2.2), (2.6). The results are shown in figure 1 for different values of the gyricity constant. The quantities $\hat{\omega }$ and $\hat{h}$ in the diagram represent the normalized eigenfrequency and the normalized gyricity, respectively, which are defined as

2.7 $$\hat{\omega }=\omega \sqrt{\frac{\rho AL^{4}}{EJ}}$$

and

2.8 $$\hat{h}=\frac{h}{\sqrt{EJ\rho A}}.$$

 Figure 1 shows the eigenfrequencies for a beam with square cross section. For $\hat{h}=0$, the eigenfrequencies have multiplicity 2; for $\hat{h}\neq 0$, each pair of eigenfrequencies splits into two, one smaller and one larger than the double eigenfrequency for $\hat{h}=0$. This phenomenon was already observed in [1] for a gyrobeam with a rectangular cross section and a non-uniform gyricity distribution. This will be exploited in §§4 and 5 to design an efficient system of resonators to reduce the vibrations of an elastic multi-structure. As the number of eigenfrequencies which accumulate in a prescribed low-frequency range becomes larger as the gyricity constant is increased, we can take a large value of the gyricity constant in order to have many eigenfrequencies in that low-frequency range. When the structure is subjected to a dynamic force whose Fourier spectrum has one or more frequencies falling inside the considered low-frequency range, the gyrobeams act as resonators because their eigenfrequencies are close to the frequencies of the external force. Consequently, they start vibrating and thus divert the energy coming from the external excitation away from the main structure which, as a result, undergoes smaller vibrations.

The first three types of eigenmodes of the clamped-free gyrobeam (denoted as modes I, II and III), calculated for $\hat{h}=1$, are illustrated in the bottom part of figure 1. The two deformed shapes associated with each mode have the same number of nodes, which are the points where the displacement is zero. However, they differ in the number and positions of the points of inflection, which are defined as the points along the beam axis where the curvature changes sign and the bending moment is zero. The locations of the inflection points are identified with small crosses in the insets of figure 1. This observation was not discussed in [1].

In order to better visualize the three-dimensional deformed shapes presented in the bottom part of figure 1, we have applied to the top of the gyrobeam a harmonic force having a frequency equal to one of the eigenfrequencies of the beam for the case $\hat{h}=1$. We have then computed the responses of the gyrobeam in the steady-state regime for the six eigenfrequencies indicated in figure 1*a*–*f*. The motion of the beam for each frequency is shown in electronic supplementary material, Video S1. There, the arrow represents the direction of the harmonic force. The trajectory of each point of the gyrobeam is a circle, since the amplitudes of *u* and *v* are identical and their phase difference is ±*π*/2. We point out that the aim of the video is only to show how the gyrobeam deforms at different frequencies.

It is interesting to observe that the curves $\sqrt{\hat{\omega }}$ versus $\sqrt{\hat{h}}$ in figure 1 intersect at a number of points where the gyrobeam possesses a double eigenfrequency. The eigenmodes associated with each double eigenfrequency are different, being characterized by a different number of nodes.

## 3. Analysis of a frame with a gyro-elastic column

**Figure 2.**
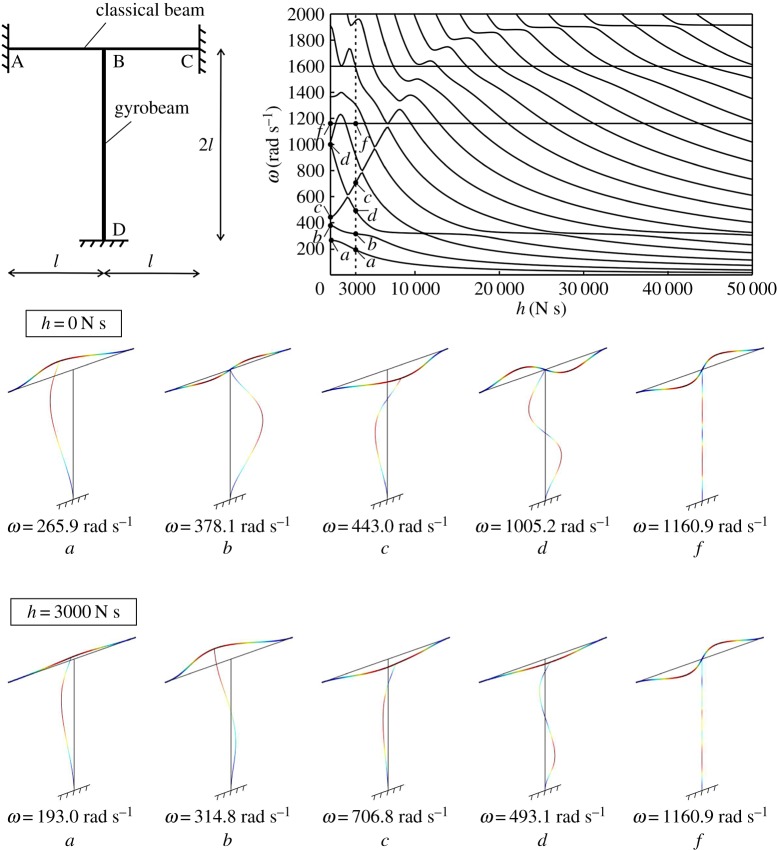
Eigenfrequencies *ω* of a structural frame made of a classical Euler–Bernoulli beam and a gyro-elastic column, for different values of the gyricity constant *h*. A comparison of some modes of the structure for *h*=0 N s and *h*=3000 N s is also shown. (Online version in colour.)

We study the structural frame sketched in figure 2, consisting of a continuous Euler–Bernoulli beam (A–C) and a column with distributed gyricity (B–D). Both ends of the horizontal beam and the bottom end of the column are clamped; the connection between the beam and the column is assumed to be rigid, which implies continuity of displacements, rotations, moments and forces at the junction. For this structure, we also take into account the longitudinal displacements of both structural elements. We assume that the two beams have length *L*=2 *l*=2 m and a square cross section of side length 0.05 m, and that they are made of steel, having Young’s modulus *E*=210 GPa, Poisson’s ratio *ν*=0.3 and density *ρ*=7850 kg m^−3^.

The eigenfrequencies *ω* of the frame are plotted in figure 2 against the gyricity constant *h*. It is apparent that gyricity affects the dynamic behaviour of the system, making it more flexible or rigid depending on the vibration mode. The most interesting feature is that the eigenfrequencies cluster at low frequencies for large values of *h*, as observed for a single gyrobeam.

The eigenmodes associated with the lowest four eigenfrequencies for the cases *h*=0 N s and *h*=3000 N s are illustrated in the bottom part of figure 2 (modes *a*–*d*). The eigenmodes were obtained by using the finite-element software Comsol Multiphysics${\;}^{®}$, while the eigenfrequencies were calculated analytically and verified with the finite-element model. It is interesting to observe how the gyricity constant modifies the deformed shapes of the frame at the different frequencies.

The motion of the gyrobeam under an external excitation is displayed in the video included in electronic supplementary material, Video S2. There, the gyricity constant is *h*=3000 N s and the frame is subjected to a harmonic force applied to the junction, acting along the *x*-direction and having a radian frequency *ω*=314.8 rad s^−1^ (which corresponds to the second eigenfrequency of the frame for that value of *h*). The video shows that the horizontal beam moves in the *y*-direction though the force acts in the *x*-direction (the *u* displacement of the junction point is very small because of the high axial stiffness of the horizontal beam). Moreover, it can be noticed from the top view of the frame that the gyro-elastic column rotates around the *z*-axis, hence its points move both in the *x*- and *y*-directions.

Finally, we observe that some lines in figure 2 are horizontal, which means that some eigenfrequencies are not affected by a variation in the gyricity constant. This occurs for the eigenmodes in which the gyro-elastic column is not deformed (see, for example, modes *f* in the bottom part of figure 2).

## 4. Floquet–Bloch waves in a periodic structure with gyrobeams

**Figure 3.**
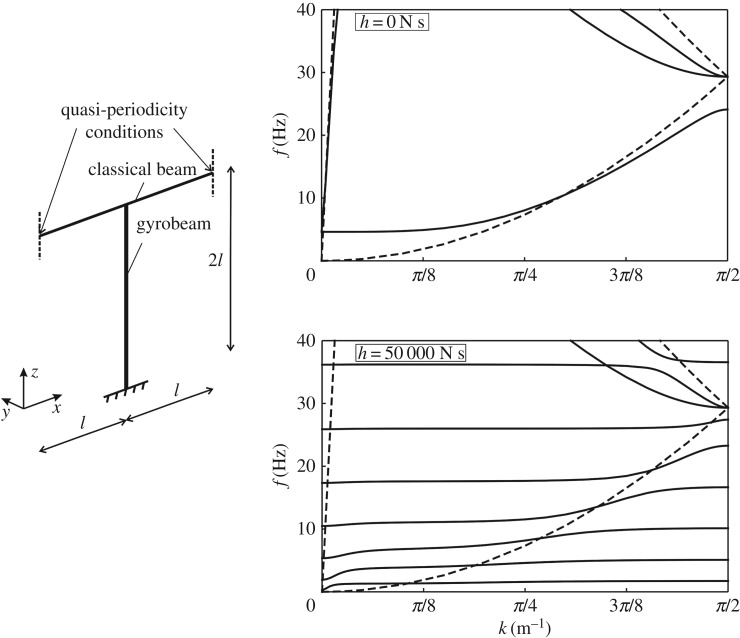
Dispersion curves for the periodic frame on the left, obtained for the following values of the gyricity constant in the column: *h*=0 N s and *h*=50 000 N s. The dashed lines are the dispersion curves for the infinite Euler–Bernoulli beam.

In this section, we consider a system made of a very large number of frames, attached to each other. One of the frames is shown in figure 3. This system can be studied as a periodic structure by imposing the quasi-periodicity (or Floquet–Bloch) conditions at the ends of a repetitive frame (or periodic cell). The quasi-periodicity conditions are expressed by

4.1 $$\Upsilon (x_{*}+L,\;y,\;z)=\Upsilon (x_{*},\;y,\;z)\;e^{ikL},$$

where x_*_ is the *x* coordinate of the left end of the horizontal beam, *L*=2 *l* is the length of the periodic cell, *k* is the wavenumber and ***Υ*** is the vector of generalized displacements and forces, given by ***Υ***=(***U***,***Φ***,***M***,***F***). Here, ***U***=(*U*,*V*,*W*) is the vector of displacements, ***Φ***=(*dU*/*dx*,*dV*/*dx*,*dW*/*dx*) is the vector of axial strain and rotations, ***M***=(*GθJ*_*t*_,*EJ*(*d*^2^*V*/*dx*^2^),*EJ*(*d*^2^*W*/*dx*^2^)) is the vector of twisting and bending moments (where *G* is the shear modulus, *θ* is the twisting angle and *J*_*t*_ is the twisting moment), and ***F***=(*EA*(*dU*/*dx*),*EJ*(*d*^3^*V*/*dx*^3^),*EJ*(*d*^3^*W*/*dx*^3^)) is the vector of axial and shear forces.

The periodic cell in figure 3 has been modelled in the finite-element software Comsol Multiphysics${\;}^{®}$, where the Floquet–Bloch conditions (4.1) have been imposed at the ends of the horizontal beam. We have considered two cases: in the first one, the gyricity constant *h*=0 N s in the entire structure; in the second one, *h*=50 000 N s in the column and *h*=0 N s in the horizontal beam. The value *h*=50 000 N s is of special interest, because for this value of the gyricity constant sufficiently many eigenfrequencies of the gyrobeam accumulate near the origin, as shown in figure 2. The dispersion curves corresponding to these two cases are plotted in figure 3, where *f*=*ω*/(2*π*) is the frequency in Hz. The dashed lines in figure 3 represent the dispersion curves for an infinite Euler–Bernoulli beam without supports, which are given in [12,35,36]

4.2 $$\begin{array}{rl} \cos\left(\beta L\right) & =\cos\left(kL\right)\;\text{with }\beta =\sqrt[{4}]{\frac{\rho A\omega^{2}}{EJ}}\;\text{(for flexural waves)}\\ \text{and}\;\cos\left(\frac{\omega L}{c}\right) & =\cos\left(kL\right)\;\text{with }c=\sqrt{\frac{E}{\rho }}\;\text{(for compressional waves)}. \end{array}\}$$

By looking at the dispersion diagram for *h*=0 N s, it can be seen that the main effect of the column is to open a zero-frequency band-gap and to generate other stop-bands at higher frequencies by decreasing the upper limits of the branches. At the boundaries of the Brillouin zone, standing waves are observed, characterized by zero group velocity. When the gyricity constant in the column is increased, more dispersion curves appear within the same frequency range, accompanied by the formation of new stop-bands. However, these stop-bands are very narrow.

**Figure 4.**
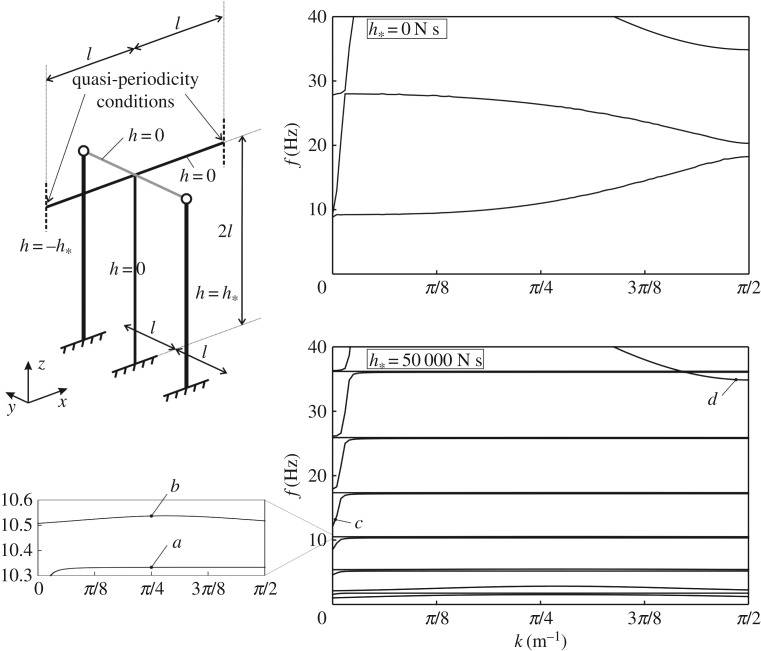
Dispersion curves for the periodic structure on the left, calculated for h_*_=0 N s and h_*_=50 000 N s.

An alternative is presented in figure 4: the main structure (namely, the horizontal beam and the column) is made of Euler–Bernoulli beams without gyricity, and two gyro-elastic columns are connected to it by means of a rigid beam (depicted by a grey line). Displacements, rotations, moments and forces are continuous at the junction between the main structure and the rigid beam. The gyricity constants of the two gyrobeams are equal in absolute value but have opposite sign, hence they rotate in opposite directions. The absolute value of the gyricity constants of the two gyrobeams is indicated by h_*_. The gyrobeams are connected to the rigid beam by hinges (drawn as empty circles), which have proved to be the best internal constraint for our purposes after a thorough study of the structure.

**Figure 5.**
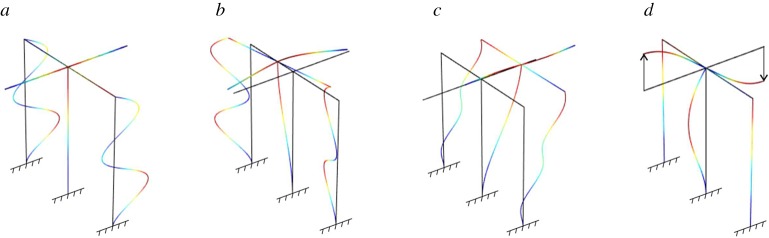
Eigenmodes of the structure in figure 4, corresponding to the eigenfrequencies indicated in figure 4. (Online version in colour.)

The dispersion curves for the cases when the entire structure has zero gyricity and when the gyrobeams have gyricity constant h_*_=±50 000 N s are shown on the right of figure 4. The eigenfrequencies determined for low values of the wavenumber *k* are mainly associated with the axial deformations of the horizontal beam (along the *x*-direction), as illustrated by the eigenmode included in figure 5*c*. These vibrations are usually not dangerous, since appropriate constraints are usually imposed at the ends of the corresponding finite structure to prevent the rigid motion in the *x*-direction and also the beams are very rigid in the axial direction. Consequently, considering only the transverse vibrations of the horizontal beam (along the *y*- and *z*-directions), it can be seen that the dispersion diagram is characterized by very narrow pass-bands at low frequencies and flat dispersion curves at higher frequencies. Accordingly, we expect that transverse waves do not propagate in such a system, except for small frequency intervals. Vibrations associated with the upper dispersion curves displayed in figure 4 are mainly in the *z*-direction, as shown by the eigenmode in figure 5*d*.

The frequencies of the flat dispersion curves can be estimated analytically. They coincide with the eigenfrequencies of a single gyrobeam with a clamped end and a pinned end, which were calculated by using the analytical approach described in §2. An example of an eigenmode of the structure in correspondence with a flat dispersion curve, shown in detail in the zoom of the dispersion diagram of figure 4, is presented in figure 5*a*. From figure 5, it is apparent that, at this frequency, the main structure does not vibrate, and all the energy of deformation is confined within the gyrobeams. Above each flat dispersion curve, there is a stop-band and, at higher frequencies, a small pass-band appears, where the entire structure vibrates (see figure 5, mode *b*). However, this pass-band is very narrow.

The dispersion curves determined for some intermediate values of h_*_ between h_*_=0 N s and h_*_=50 000 N s are presented in appendix A.

The structural configuration sketched in figure 4 will be used in the next section to show how gyrobeams can be employed to reduce the vibrations of the main horizontal beam when the structure is subjected to an external excitation.

## 5. Transmission problem in a multi-structure with gyrobeams

**Figure 6.**
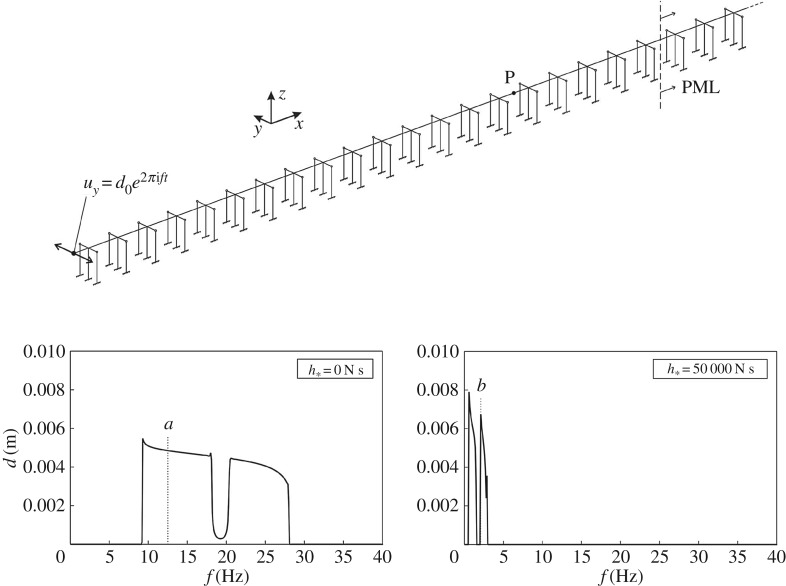
Response in the frequency domain of a semi-infinite structure, shown at the top, for h_*_=0 N s (left diagram) and h_*_= 50 000 N s (right diagram). The displacement of point P due to a harmonic displacement of amplitude *d*_0_=0.01 m and variable frequency *f* is plotted in both diagrams. PML are added to prevent reflections of waves at the right boundary.

In order to test the isolation device proposed in §4, we study the structure illustrated in the top part of figure 6, which consists of an assembly of many repetitive units, as that depicted in figure 4. At the left end of the structure we impose a harmonic displacement of amplitude *d*_0_ and frequency *f*, acting in the *y*-direction. At the right end, we introduce PMLs (perfectly matched layers), which are used to minimize reflections of waves at the boundary and to model a semi-infinite system. The PML are designed to gradually dissipate the energy of the impinging waves [37]. The absorption effect is introduced by implementing a complex Young’s modulus in the beams, given by

5.1 $$E_{c}=E(1+i\eta ),\;\text{with}\;\eta =\eta_{1}[e^{\eta_{2}(x-x_{\mathrm{PML}})}-1].$$

In the formula above, *η* is the *absorption factor* or *isotropic loss factor*, and *x*_PML_ is the *x* coordinate of the interface between the non-dissipating frames and the PML. The coefficients *η*_1_ and *η*_2_ are tuned to make the absorption coefficient increase slowly, since a high-contrast interface would generate reflected waves. The part of the structure with PML needs to be long enough to dissipate all the energy travelling through it. An alternative type of PML in a flexural system is discussed in [38].

**Figure 7.**
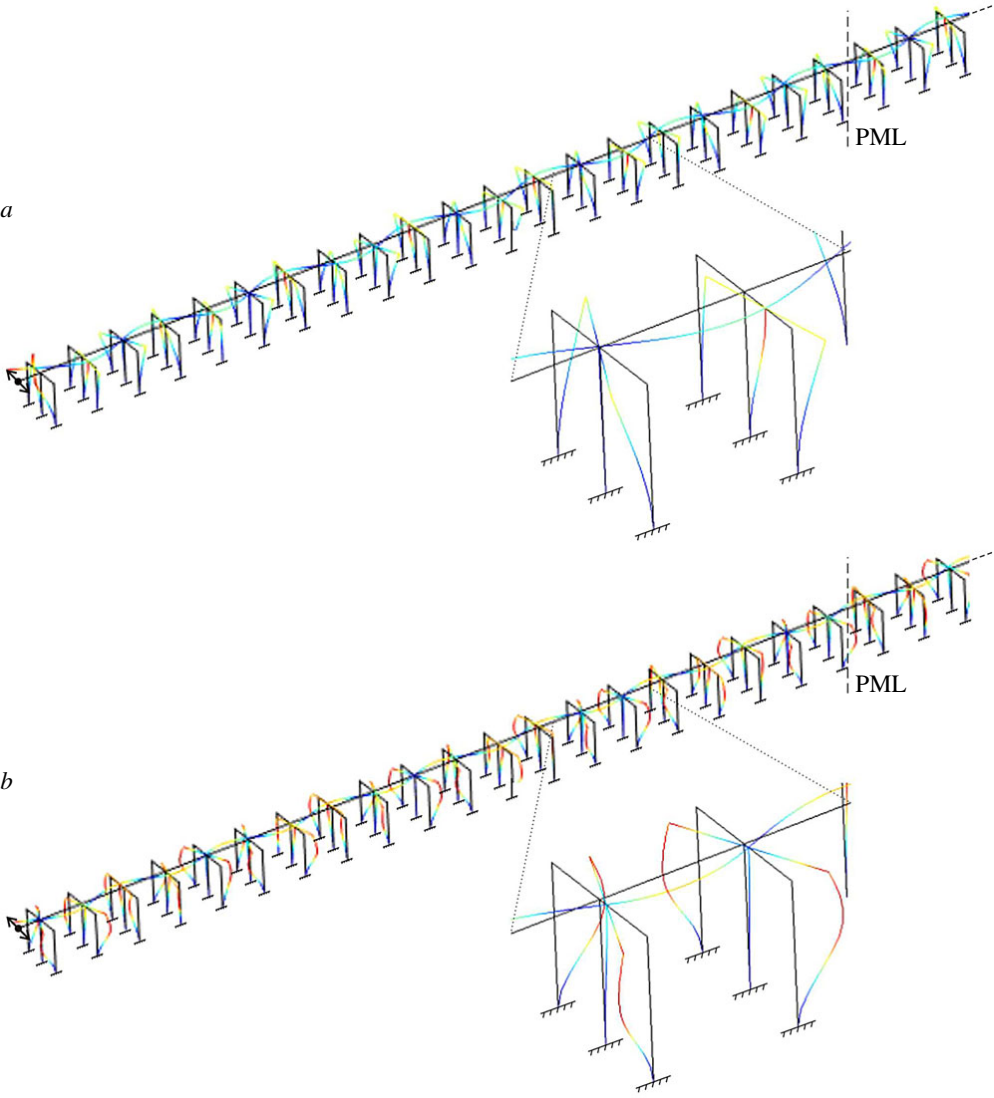
Deformed shapes of the structure in figure 6, computed at the frequencies indicated in figure 6. (Online version in colour.)

We have built a finite-element model in Comsol Multiphysics${\;}^{®}$ to determine the displacement field in the structure produced by the imposed displacement. The computations have been performed in the frequency domain. As earlier, we have examined two scenarios: in the first one, the entire structure is made of classical Euler–Bernoulli beams, while in the second one gyricity is introduced in the lateral columns, which have gyricity constant ±h_*_ (the reader is referred to the periodic unit of figure 4 for the position of the gyrobeams in each frame). In both cases, the beams connecting the gyrobeams to the main structure are rigid. Figure 6 shows the displacement amplitude of a point of the structure, indicated by P in the top part of the figure, for both scenarios. Additional computations have shown that very similar diagrams are found for the other points of the structure, provided that they are not too close to the external excitation. These results are included in electronic supplementary material, Fig. S6. The deformed shapes of the system in the two scenarios are presented in figure 7 for two different frequencies, at which waves propagate.

The displacement amplitudes plotted in figure 6 are different from zero at the frequencies within the pass-bands of the corresponding periodic structure, determined from figure 4. This shows that the forced response of the semi-infinite structure can be deduced from the behaviour of the infinite system, represented by the dispersion diagram. The very narrow pass-bands calculated for the system with gyrobeams at higher frequencies were not detected by the model in the frequency domain, though the frequency step adopted in the computations was very small (Δ*f*=0.0001 Hz).

The most important outcome of figure 6 is that the frequency ranges where waves can propagate are reduced significantly if gyrobeams are employed. This demonstrates that gyrobeams are an efficient tool to mitigate the transverse vibrations of a structural system if the frequency of the applied force is not very low. An enhanced design, combining alternating frames with and without gyrobeams, is discussed below.

In the numerical computations, we have not considered an external excitation acting along the *z*-direction, because the ensuing waves would not trigger the gyricity effect in the columns. Indeed, the dispersion curves corresponding to this type of waves are not altered by changing the gyricity constant in the columns (compare the upper dispersion curves in figure 4 for h_*_=0 N s and h_*_=50 000 N s). On the other hand, an external excitation acting in the *x*-direction would generate compressional and extensional waves in the horizontal beams which, however, occur at higher frequencies due to the high longitudinal stiffness of the beams. In addition, rigid motions along the *x*-direction are usually prevented in practice by introducing appropriate constraints (e.g. hinges) at the abutments of the structure.

The structural responses of the system for other values of the gyricity constant are included in appendix B.

### (a) Effect of damping on the dynamic response

We have also computed the response of the semi-infinite system shown in the top part of figure 6 when damping is introduced in the lateral columns of each frame (i.e. the thick lines in the periodic unit of figure 4). Damping was introduced by defining Young’s modulus of all lateral columns as

5.2 $$E_{c}=E(1+i\eta ).$$

We have considered a uniform absorption factor equal to 5%, namely *η*=0.05, which is very common in civil engineering structures [13].

**Figure 8.**
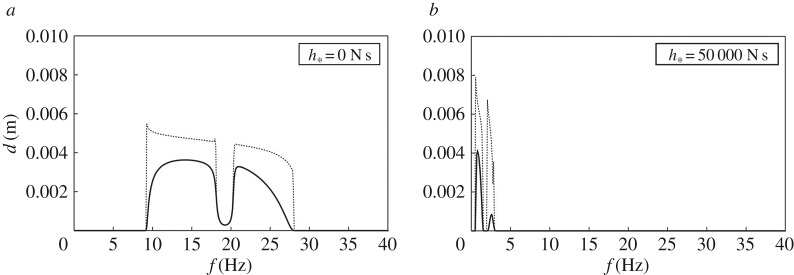
Same as in figure 6, but after adding damping to the lateral columns (without and with gyricity). The dotted lines represent the responses of the system without damping, taken from figure 6.

The structural response of the system for different values of the frequency is plotted in figure 8 for the cases where the lateral columns are classical Euler–Bernoulli beams and gyrobeams. To simplify the comparison with figure 6, we have also shown in figure 8 the displacement amplitudes determined without damping (dotted lines). It can be noticed from the figure that the main advantage of adding damping to the structure is obtained when h_*_≠0, because in this case the displacement amplitudes decrease significantly.

### (b) Enhanced design of a multi-structure

**Figure 9.**
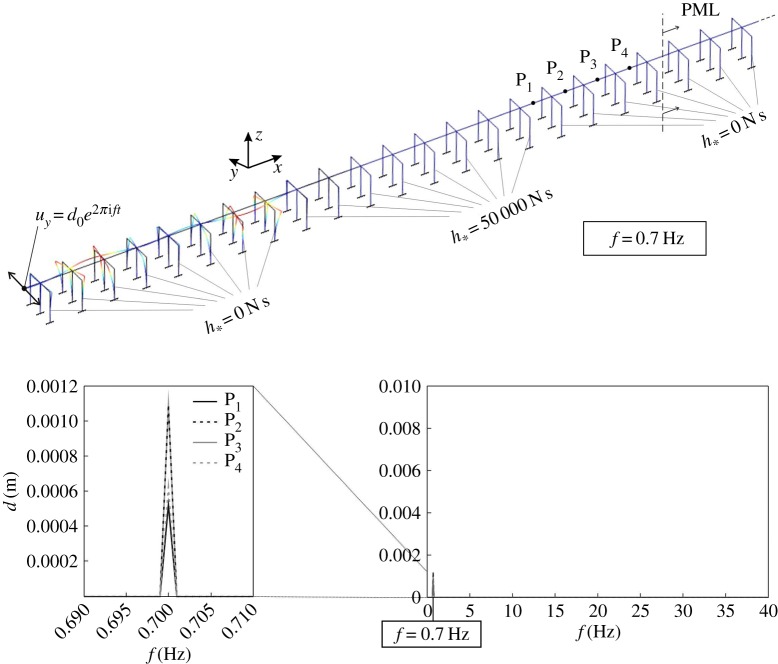
Displacement amplitudes, produced by an external excitation, computed at four different points of a semi-infinite structure, consisting of an alternating series of frames with and without gyricity. The deformed shape of the structure determined at *f*=0.7 Hz is illustrated at the top. (Online version in colour.)

We have shown that a periodic system made of an infinite array of frames as that of figure 4 creates a low-frequency band-gap when h_*_=0 N s and a large band-gap at higher frequencies when h_*_=50 000 N s. Accordingly, we can create a very efficient filter for transverse waves if we design the structure as an alternating series of frames with and without gyricity. An example of such a structure is depicted in the top part of figure 9. It consists of an array of eight frames without gyricity and eight frames with gyricity, and it is modelled as a semi-infinite system by inserting PML.

We again apply a harmonic displacement at the left end of the structure and we compute the resulting displacement field. In figure 9, we plot the displacement amplitudes determined at four different points, indicated by P_1_–P_4_, which are located after 16 frames, namely after one set of eight frames without gyricity and one set of eight frames with gyricity. The diagram reveals that, apart from a small peak at *f*=0.7 Hz, the displacement amplitudes are negligible, implying that waves cannot travel in this structure. Further computations have shown that, if the number of frames in each set is reduced, the frequency intervals where waves can propagate are increased.

The deformed shape of the structure at *f*=0.7 Hz is illustrated in the top part of figure 9. It is apparent that the displacement amplitudes are sizable only in the first set of frames with h_*_=0 N s, where they are given as the superposition of the incident waves and the waves reflected at the interface between the two sets of eight frames. In the second set of frames, where h_*_=50 000 N s, the displacement amplitudes are much smaller, because *f*=0.7 Hz is within the stop-band of the structure with zero gyricity.

## 6. Conclusion

A highly efficient wave filtering system, whose design includes gyro-elastic beam resonators, has been proposed in this paper. It has been demonstrated that gyrobeams provide a new tuning mechanism, different from the known high-contrast resonators or conventional tuned mass dampers.

The analysis of the dynamic response of a single gyrobeam has shown that a large number of eigenfrequencies tend to cluster within a low-frequency interval as the gyricity constant is increased. In a periodic system made of an infinite array of elastic gyroscopic frames, gyro-elastic beams contribute to very interesting new dispersion properties of waves and, in particular, standing modes. We have proposed a novel design, in which two systems of gyrobeams rotating with the same speed and in opposite directions are attached to the main structure, such as a bridge. In this case, very narrow pass-bands corresponding to transverse modes have been observed from the dispersion analysis. Accordingly, when determining the response of the same system under an external excitation, we have found that the frequency intervals where waves propagate are reduced significantly.

As discussed in §5a, dissipative structures are very important for the reduction of vibrations initiated by seismic loads. We have also demonstrated in this paper that the role of gyrobeams is primarily to create low-frequency ‘energy sinks’, in which waves generated by external excitations are channelled. As a consequence, energy is diverted away from the main structure, which undergoes smaller displacements and smaller stresses. Additional dampers may then be attached to the gyrobeams to reduce their vibrations.

Gyrobeams offer a practical alternative to methods currently used to reduce the effects caused by seismic waves. This work opens a new perspective in chiral metamaterial design and in a wide range of applications in earthquake wave filtering.

## Supplementary Material

Frequency response of a semi-infinite structure made of repeating frames

## Figures and Tables

**Figure 10.**
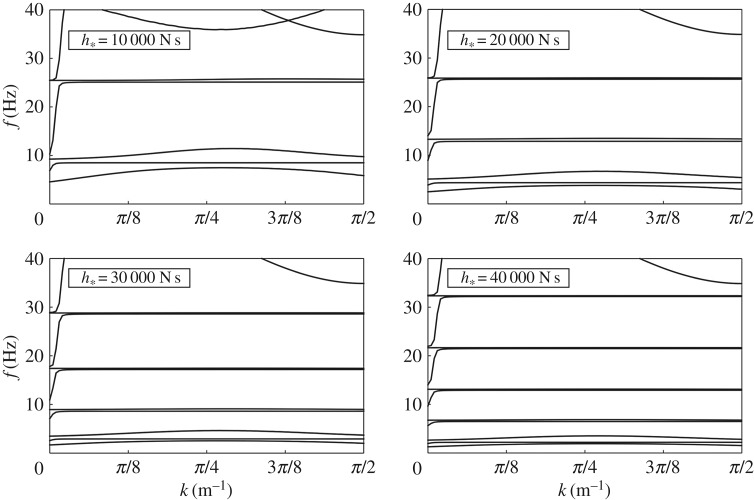
Dispersion curves for the periodic frame in figure 4, determined for different values of the gyricity constant h_*_.

**Figure 11.**
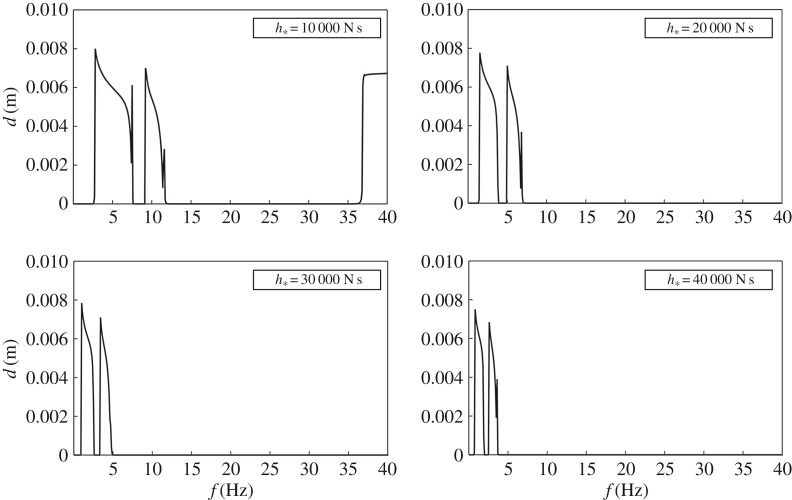
Displacement amplitude of a point of a semi-infinite structure (point P in figure 6), due to an externally imposed harmonic displacement, determined for different values of the gyricity constant h_*_.

## Data accessibility

This paper contains no experimental data. All computational results are reproducible.

## Appendix A. Dispersion curves for other values of the gyricity constant

In figure 10, we present the dispersion curves relative to the structural frame with quasi-periodicity conditions shown in figure 4, calculated for the following values of the gyricity constant: *h*_*_=10 000 N s, *h*_*_=20 000 N s, *h*_*_=30 000 N s and *h*_*_=40 000 N s. By comparing the diagrams in figure 10, it can be seen that the number of dispersion curves increases and the low-frequency pass-bands shrink as the value of the gyricity constant is increased.

## Appendix B. Structural response in the frequency domain for other values of the gyricity constant

We plot in figure 11, the displacement amplitudes of the point P of the semi-infinite system shown in the top part of figure 6, produced by a harmonic displacement of amplitude *d*_0_=0.01 m and frequency *f* applied at the left end of the system, computed for the following values of the gyricity constant: *h*_*_=10 000 N s, *h*_*_=20 000 N s, *h*_*_=30 000 N s and *h*_*_=40 000 N s. As the value of *h*_*_ is increased, the low-frequency propagation ranges are reduced in size and move to lower frequencies. Moreover, the high-frequency propagation range observed for the case *h*_*_=10 000 N s disappears for higher values of *h*_*_, because it is shifted to higher frequencies.

## References

[1] D’Eleuterio GMT, Hughes PC 1984 Dynamics of gyroelastic continua. *J. Appl. Mech.* 51, 415–422. (doi:10.1115/1.3167634)

[2] Mead DJ 1970 Free wave propagation in periodically supported, infinite beams. *J. Sound Vib.* 11, 181–197. (doi:10.1016/S0022-460X(70)80062-1)

[3] SenGupta G 1970 Natural flexural waves and the normal modes of periodically-supported beams and plates. *J. Sound Vib.* 13, 89–101. (doi:10.1016/S0022-460X(70)80082-7)

[4] SenGupta G 1971 Natural frequencies of periodic skin-stringer structures using a wave approach. *J. Sound Vib.* 16, 567–580. (doi:10.1016/0022-460X(71)90663-8)

[5] Mead DJ 1975 Wave propagation and natural modes in periodic systems: II. Multi-coupled systems, with and without damping. *J. Sound Vib.* 40, 19–39. (doi:10.1016/S0022-460X(75)80228-8)

[6] Mead DJ 1996 Wave propagation in continuous periodic structures: research contributions from Southampton, 1964–1995. *J. Sound Vib.* 190, 495–524. (doi:10.1006/jsvi.1996.0076)

[7] Heckl MA 2002 Coupled waves on a periodically supported Timoshenko beam. *J. Sound Vib.* 252, 849–882. (doi:10.1006/jsvi.2001.3823)

[8] Romeo F, Luongo A 2002 Invariants representation of propagation properties for bi-coupled periodic structures. *J. Sound Vib.* 257, 869–886. (doi:10.1006/jsvi.2002.5065)

[9] Brun M, Giaccu GF, Movchan AB, Movchan NV 2011 Asymptotics of eigenfrequencies in the dynamic response of elongated multi-structures. *Proc. R. Soc. A* 468, 378–394. (doi:10.1098/rspa.2011.0415)

[10] Carta G, Brun M, Movchan AB 2014 Dynamic response and localization in strongly damaged waveguides. *Proc. R. Soc. A* 470, 20140136 (doi:10.1098/rspa.2014.0136)

[11] Carta G, Brun M, Movchan AB 2014 Elastic wave propagation and stop-band generation in strongly damaged solids. *Fract. Struct. Int.* 29, 28–36. (doi:10.3221/IGF-ESIS.29.04)

[12] Carta G, Brun M 2015 Bloch-Floquet waves in flexural systems with continuous and discrete elements. *Mech. Mat.* 87, 11–26. (doi:10.1016/j.mechmat.2015.03.004)

[13] Carta G, Movchan AB, Argani LP, Bursi OS 2016 Quasi-periodicity and multi-scale resonators for the reduction of seismic vibrations in fluid-solid systems. *Int. J. Eng. Sci.* 109, 216–239. (doi:10.1016/j.ijengsci.2016.09.010)

[14] Carta G, Brun M, Movchan AB, Boiko T 2016 Transmission and localisation in ordered and randomly-perturbed structured flexural systems. *Int. J. Eng. Sci.* 98, 126–152. (doi:10.1016/j.ijengsci.2015.09.005)

[15] Jones IS, Movchan AB, Gei M 2011 Waves and damage in structured solids with multi-scale resonators. *Proc. R. Soc. A* 467, 964–984. (doi:10.1098/rspa.2010.0319)

[16] Bigoni D, Guenneau S, Movchan AB, Brun M 2013 Elastic metamaterials with inertial locally resonant structures: application to lensing and localization. *Phys. Rev. B* 87, 174303 (doi:10.1103/PhysRevB.87.174303)

[17] Tallarico D, Movchan NV, Movchan AB, Colquitt DJ 2016 Tilted resonators in a triangular elastic lattice: Chirality, Bloch waves and negative refraction. *J. Mech. Phys. Solids* 103, 236–256. (doi:10.1016/j.jmps.2017.03.007)

[18] Achaoui Y, Ungureanu B, Enoch S, Brûlé S, Guenneau S 2016 Seismic waves damping with arrays of inertial resonators. *Extr. Mech. Lett.* 8, 30–37. (doi:10.1016/j.eml.2016.02.004)

[19] Colombi A, Colquitt D, Roux P, Guenneau S, Craster RV 2016 A seismic metamaterial: the resonant metawedge. *Sci. Rep.* 6, 27717 (doi:10.1038/srep27717) 27283587 10.1038/srep27717 PMC4901369

[20] Meirovitch L 1974 A new method of solution of the eigenvalue problem for gyroscopic systems. *AIAA J.* 12, 1337–1342. (doi:10.2514/3.49486)

[21] Meirovitch L 1975 A modal analysis for the response of linear gyroscopic systems. *J. Appl. Mech.* 42, 446–450. (doi:10.1115/1.3423597)

[22] Huseyin K, Plaut RH 1974 Transverse vibrations and stability of systems with gyroscopic forces. *J. Struct. Mech.* 3, 163–177. (doi:10.1080/03601217408907262)

[23] Huseyin K 1976 Standard forms of the eigenvalue problems associated with gyroscopic systems. *J. Sound Vib.* 45, 29–37. (doi:10.1016/0022-460X(76)90665-9)

[24] Inman DJ 1988 A sufficient condition for the stability of conservative gyroscopic systems. *J. Appl. Mech.* 55, 895–898. (doi:10.1115/1.3173738)

[25] Huseyin K 1991 On the stability criteria for conservative gyroscopic systems. *J. Vib. Acoust.* 113, 58–61. (doi:10.1115/1.2930155)

[26] Thomson W 1894 *The molecular tactics of a crystal*. Oxford, UK: Clarendon Press.

[27] Brun M, Jones IS, Movchan AB 2012 Vortex-type elastic structured media and dynamic shielding. *Proc. R. Soc. A* 468, 3027–3046. (doi:10.1098/rspa.2012.0165)

[28] Carta G, Brun M, Movchan AB, Movchan NV, Jones IS 2014 Dispersion properties of vortex-type monatomic lattices. *Int. J. Solids Struct.* 51, 2213–2225. (doi:10.1016/j.ijsolstr.2014.02.026)

[29] Wang P, Lu L, Bertoldi K 2015 Topological phononic crystals with one-way elastic edge waves. *Phys. Rev. Lett.* 115, 104302 (doi:10.1103/PhysRevLett.115.104302) 26382680 10.1103/PhysRevLett.115.104302

[30] Nash LM, Kleckner D, Read A, Vitelli V, Turner AM, Irvine WTM 2015 Topological mechanics of gyroscopic metamaterials. *Proc. Natl Acad. Sci. USA* 112, 14 495–14 500. (doi:10.1073/pnas.1507413112) 26561580 10.1073/pnas.1507413112 PMC4664354

[31] Carta G, Jones IS, Movchan NV, Movchan AB, Nieves MJ 2017 ‘Deflecting elastic prism’ and unidirectional localisation for waves in chiral elastic systems. *Sci. Rep.* 7, 26 (doi:10.1038/s41598-017-00054-6) 28154420 10.1038/s41598-017-00054-6 PMC5428365

[32] Hughes PC, D’Eleuterio GMT 1986 Modal parameter analysis of gyroelastic continua. *J. Appl. Mech.* 53, 918–924. (doi:10.1115/1.3171881)

[33] D’Eleuterio GMT 1988 On the theory of gyroelasticity. *J. Appl. Mech.* 55, 488–489. (doi:10.1115/1.3173705)

[34] Yamanaka K, Heppler GR, Huseyin K 1996 Stability of gyroelastic beams. *AIAA J.* 34, 1270–1278. (doi:10.2514/3.13223)

[35] Graff KF 1991 *Wave motion in elastic solids*. New York, NY: Dover.

[36] Brun M, Guenneau S, Movchan AB, Bigoni D 2010 Dynamics of structural interfaces: filtering and focussing effects for elastic waves. *J. Mech. Phys. Solids* 58, 1212–1224. (doi:10.1016/j.jmps.2010.06.008)

[37] Carta G, Jones IS, Brun M, Movchan NV, Movchan AB 2013 Crack propagation induced by thermal shocks in structured media. *Int. J. Solids Struct.* 50, 2725–2736. (doi:10.1016/j.ijsolstr.2013.05.001)

[38] Morvaridi M, Brun M 2016 Perfectly matched layers for flexural waves: an exact analytical model. *Int. J. Solids Struct.* 102–103, 1–9. (doi:10.1016/j.ijsolstr.2016.10.024)

