# Supplementary material for: Gyro-elastic beams for the vibration reduction of long flexural systems
Source: Proc Math Phys Eng Sci. 2017 Jul 19;473(2203):20170136. doi: 10.1098/rspa.2017.0136 (PMC5549565; doi:10.1098/rspa.2017.0136)
Supplement: Frequency response of a semi-infinite structure made of repeating frames [file rspa20170136supp1.pdf]

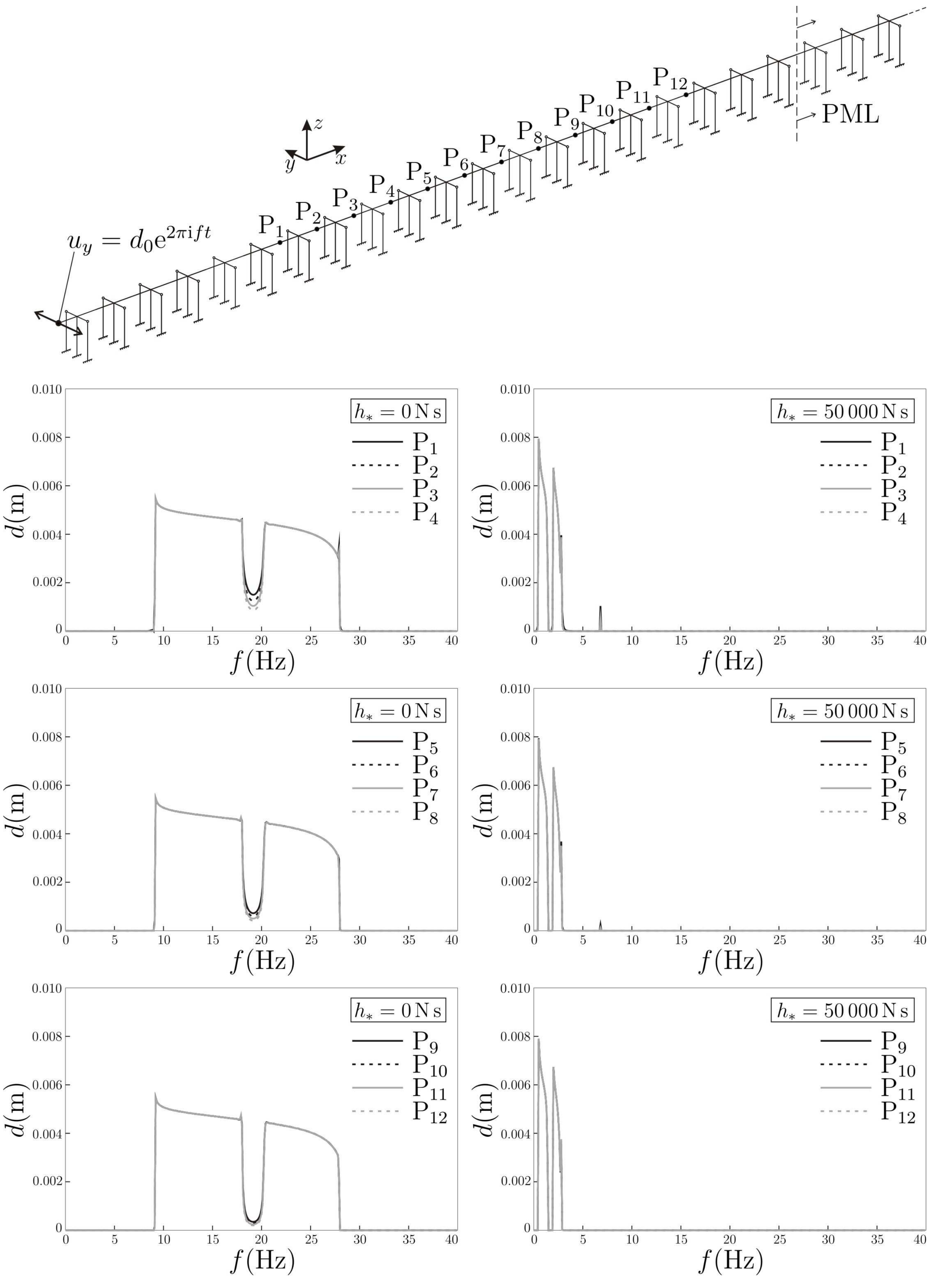

As in Fig. 6 of the paper, but for different points of the structure, indicated at the top. We note that in the diagrams on the right the responses of the structure at different points are very similar, hence the curves almost coincide.
